# Association between A Body Shape Index with constipation and diarrhea: NHANES 2005 to 2010

**DOI:** 10.1097/MD.0000000000050446

**Published:** 2026-09-04

**Authors:** Canjie Wei, Yangtao Chen, Zhaochu Wang, Yameng Xie, Xun Wang, Hongling Huang, Jing Wang

**Affiliations:** a Fujian University of Traditional Chinese Medicine, Fuzhou, China; b Department of Anorectal Surgery, The Affiliated People’s Hospital of Fujian University of Traditional Chinese Medicine, Fuzhou, China.

**Keywords:** Body Shape Index, constipation, NHANES

## Abstract

A Body Shape Index (ABSI) is a composite metric designed to more accurately assess an individual’s body size and associated health risks. This study aimed to explore the potential relationship between ABSI and constipation. A total of 11,336 participants from the National Health and Nutrition Examination Survey 2005 to 2010 were included. Multivariable logistic regression models were used to evaluate the association between ABSI and constipation after adjustment for potential confounders. Restricted cubic spline analysis was performed to explore potential nonlinear associations. Subgroup and interaction analyses were conducted to assess whether the association differed across demographic and clinical characteristics. The multivariate logistic regression analysis, fully adjusted for confounders, indicated that an increase in ABSI was associated with a decrease in the incidence of constipation when ABSI was below 0.082 (OR = 5.88, 95% CI: 2.17–9.59, *P* < .01). Subgroup analysis and interaction tests revealed a significant positive association between ABSI and constipation, particularly in males, individuals with a college education or higher, alcohol users, as well as those with hypertension and diabetes. This study identified a relationship between ABSI and the incidence of constipation, suggesting that a reduction in ABSI, within specific limits, is linked to a decreased risk of constipation.

## 1. Introduction

Constipation is a prevalent gastrointestinal disorder, affecting approximately 16% of the global population, with significant variations across different demographic groups.^[1,2]^ Symptoms such as infrequent bowel movements, hard stools, and straining during defecation can lead to physical discomfort and pain. These symptoms often reduce mobility and increase fatigue, limiting daily activities and overall well-being. Moreover, the psychological impact of constipation, including frustration, embarrassment, and anxiety, is notable, especially when it affects social interactions and work productivity.^[3]^ Thus, constipation is not a minor inconvenience but a condition that can significantly affect various aspects of an individual’s life. Effective management of constipation is vital for improving both physical health and psychological well-being, thereby enhancing overall quality of life.

Research has shown that individuals with obesity are more likely to experience constipation,^[4]^ potentially due to endocrine dysregulation, inflammatory responses, and compromised gastrointestinal motility associated with obesity.^[5]^ While traditional risk factors for constipation, such as insufficient dietary fiber intake, low physical activity, and certain medications, have been extensively studied, the role of body composition in the development of constipation remains inadequately understood.

Body mass index (BMI) is the most commonly used measure of obesity, as it normalizes weight relative to height. However, BMI has notable limitations, particularly in its inability to account for body shape variation and regional fat distribution.^[6]^ To address these shortcomings, A Body Shape Index (ABSI) was developed as a novel anthropometric measure. ABSI incorporates waist circumference (WC) while adjusting for height and weight, providing a more comprehensive evaluation of body composition.^[7–9]^ Despite its potential advantages in assessing health risks, the relationship between ABSI and constipation remains largely unexplored.

The National Health and Nutrition Examination Survey (NHANES) presents a unique opportunity to examine the association between ABSI and constipation in a large, nationally representative sample of the U.S. population. The extensive data available in NHANES allows for robust adjustments of potential confounders and detailed subgroup analyses.^[10]^ Therefore, this study aims to investigate the relationship between ABSI and constipation, with a focus on potential effect modifications by demographic and clinical characteristics. Our findings may offer new insights into the role of body shape in constipation risk and guide more targeted preventive strategies.

## 2. Methods

### 2.1. Data source and participants

The NHANES is a population-based, cross-sectional research program conducted by the National Center for Health Statistics of the Centers for Disease Control and Prevention.^[11]^ Since its inception in the early 1960s, NHANES has provided valuable data, becoming an ongoing survey since 1999. The survey collects comprehensive demographic information, dietary intake data, health-related responses, medical examinations, physiological measurements, and laboratory test results through a combination of interviews and physical exams. These data are crucial for assessing the prevalence and risk factors of diseases, and for informing public health policies and health service programs. The study was approved by the Research Ethics Review Board of National Center for Health Statistics, and written informed consent was obtained from all participants.

**Figure 1.**
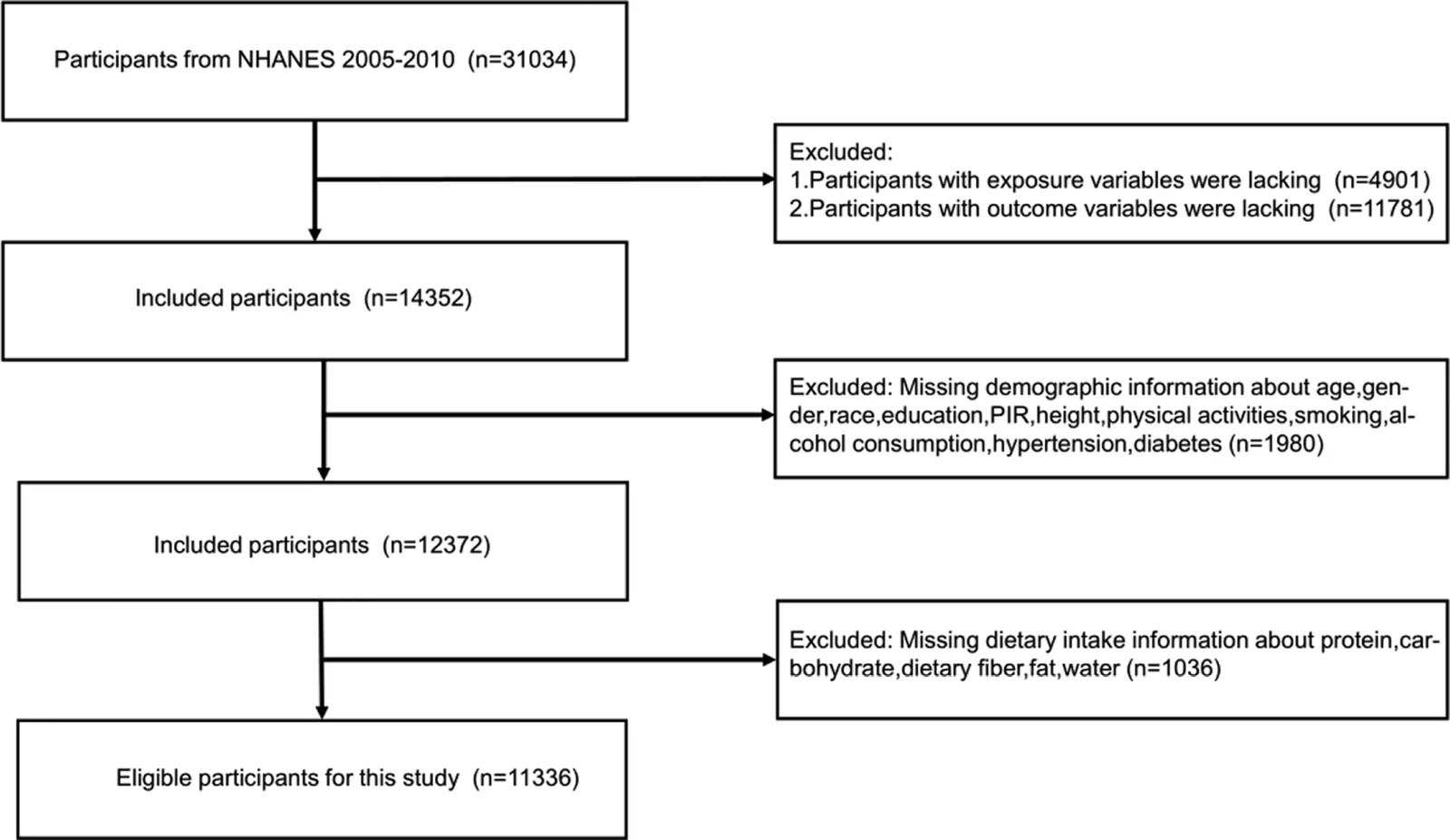
Flowchart of participant selection from NHANES 2005–2010. NHANES = Nutrition Examination Survey.

We used publicly available bowel health data from the NHANES 2005 to 2010 cycle. Participants younger than 20 years were excluded because the bowel health questionnaire was administered only to adults. Participants with missing exposure variables (ABSI), missing outcome data on bowel movement frequency, missing demographic or clinical covariates, or missing dietary intake information were further excluded. After applying these exclusion criteria, a total of 11,336 participants were included in the final analysis. The participant selection process is shown in Figure 1.

### 2.2. Index calculation

ABSI is a composite metric that combines height, weight, and WC to offer a more precise assessment of body shape and associated health risks. Higher ABSI values indicate a larger WC relative to height and weight, which is more indicative of visceral obesity. ABSI is positively correlated with visceral adiposity and has been shown to be a better predictor of premature mortality risk compared to WC and BMI. The ABSI is calculated using the following formula:


$$ABSI=\frac{WC}{BMI^{2/3}\times Height^{1/2}}$$

### 2.3. Definitions

The NHANES Bowel Health Questionnaire, which specifically assesses participants’ bowel health, includes questions about common bowel symptoms such as constipation, diarrhea, and urgency to pass stools, along with their frequency and duration. The questionnaire also assessed stool characteristics using the Bristol Stool Form Scale, a seven-category visual scale illustrated with vividly colored cards depicting stool forms ranging from Type 1 (separate hard lumps) to Type 7 (watery stool). Since stool consistency and bowel movement frequency reflect different aspects of bowel function, constipation was defined according to bowel movement frequency, consistent with a previously published study.^[12]^ Participants were asked, “How many times per week do you usually have a bowel movement?” Constipation was defined as fewer than 3 bowel movements per week, normal bowel as 3 to 21 bowel movements per week, and diarrhea as more than 21 bowel movements per week.

### 2.4. Covariates

A comprehensive set of covariates was included in this study to account for potential confounding variables. These covariates included age, gender, race, education level, poverty-to-income ratio (PIR), height, alcohol consumption, smoking status, history of diabetes, history of hypertension, vigorous physical activity, and dietary intake (including protein, carbohydrate, fiber, total fat, and water intake). Ethnicity and race were categorized into 5 groups: Mexican Americans, Other Hispanic, non-Hispanic White, non-Hispanic Black, and other ethnicities. Education level was divided into the following categories: <9th grade, 9th to 11th grade, high school graduate, some college, and college graduate or above.

Vigorous physical activity was defined as activities that cause large increases in breathing or heart rate (e.g., carrying or lifting heavy loads, digging, or construction work) performed for at least 10 continuous minutes. Smoking status was categorized into 3 groups: current smokers, former smokers, and never smokers. “Never smokers” were defined as individuals who had never smoked or had smoked fewer than 100 cigarettes before the interview. “Former smokers” were those who had smoked more than 100 cigarettes in their lifetime but had quit by the time of the interview. “Current smokers” were those who had smoked at least 100 cigarettes in their lifetime and were still smoking at the time of the interview.^[13]^

A drinker was defined as an individual who consumes at least 12 alcoholic beverages per year. Individuals with diabetes and hypertension were defined as those who had been diagnosed by a physician.

During the 2005 to 2010 NHANES cycle, a 24-hour dietary recall survey was conducted to collect data on participants’ intake of dietary fiber, water, total fat, carbohydrates, and protein. These factors were included as covariates in the analysis to more precisely assess the independent association between ABSI and constipation.

## 3. Statistical analysis

To account for the complex sampling design of NHANES, we applied appropriate weights to ensure the accuracy and representativeness of the results. Descriptive analyses were conducted using Empower Stats (version 2.0) and R software (version 4.1.3). Categorical data are presented as percentages, while continuous data are reported as either mean and standard deviation or median and interquartile range, as appropriate. The *t*-test was used for continuous variables, and the chi-square test was applied to categorical variables.

The association between ABSI and constipation was assessed using multivariate logistic regression analysis. Based on bowel movement frequency, participants were categorized into 3 groups, and trends across these groups were analyzed. Model 1 was unadjusted, while Model 2 was adjusted for age, gender, and race. Model 3 was fully adjusted for all potential confounders, including age, gender, race, education level, PIR, height, alcohol consumption, smoking status, diabetes history, hypertension history, vigorous physical activity, protein intake, carbohydrate intake, dietary fiber intake, total fat intake, and water intake.

To further explore the potential linear or non-linear relationship between ABSI and constipation, we constructed restricted cubic spline (RCS) curves. If a non-linear relationship was identified, we aimed to determine the inflection point of ABSI – the value at which a change in the curve was observed.

All analyses were performed using Empower Stats (version 2.0) and R software (version 4.1.3), with a significance threshold set at *P* < .05.

## 4. Results

### 4.1. Demographic characteristics

A total of 11,336 individuals aged 20 years and older were included in this study. Table 1 presents the characteristics of the study population, stratified according to the frequency of bowel movements, using data from the 2005 to 2010 NHANES. Among the participants, 391 reported constipation, and the subjects were categorized into 3 groups based on bowel movement frequency. In these groups, factors such as age, gender, PIR, ethnicity, BMI, diabetes mellitus, hypertension, smoking behaviors, and alcohol use were statistically significant (all *P* < .05).

**Table 1:** Characteristics of the study population.

	Constipation (n = 391)	Normal bowel (n = 10198)	Diarrhea (n = 747)	*P* value
Age	42.15 ± 15.83	46.18 ± 16.17	47.07 ± 15.17	<.0001
ABSI	0.01 ± 0.00	0.01 ± 0.00	0.01 ± 0.00	.0013
PIR	2.55 ± 1.69	3.19 ± 1.60	3.01 ± 1.65	<.0001
BMI (kg/m^2^)	27.75 ± 6.12	28.56 ± 6.48	30.33 ± 6.61	<.0001
Weight (kg)	75.67 ± 17.44	82.67 ± 20.98	88.14 ± 20.82	<.0001
Height (cm)	165.10 ± 8.38	169.86 ± 9.86	170.37 ± 9.63	<.0001
Waist circumference (cm)	94.12 ± 14.67	98.13 ± 16.12	102.88 ± 15.93	<.0001
Protein intake (g)	66.54 ± 33.89	86.27 ± 43.44	97.54 ± 54.55	<.0001
Carbohydrate intake (m)	239.76 ± 113.68	266.23 ± 129.95	285.54 ± 143.65	<.0001
Dietary fiber intake (g)	12.48 ± 7.42	16.44 ± 9.78	17.38 ± 10.48	<.0001
Total fat intake (g)	72.35 ± 40.57	85.62 ± 47.65	95.43 ± 57.70	<.0001
Moisture intake (g)	2623.11 ± 1461.49	3170.75 ± 1536.03	3566.13 ± 1662.92	<.0001
Gender				
Male	61 (15.56%)	5273 (51.71%)	472 (63.21%)	<.0001
Female	330 (84.44%)	4925 (48.29%)	275 (36.79%)	
Race				
Mexican American	20 (5.25%)	760 (7.45%)	87 (11.60%)	<.0001
Other Hispanic	16 (3.98%)	396 (3.88%)	309 (4.14%)	
Non-Hispanic White	261 (66.67%)	7591 (74.44%)	497 (66.47%)	
Non-Hispanic Black	80 (20.34%)	970 (9.51%)	94 (12.64%)	
Other Race	15 (3.76%)	481 (4.72%)	38 (5.15%)	
Education				
<9th grade	19 (4.93%)	496 (4.86%)	465 (6.22%)	<.0001
9–11th grade	65 (16.56%)	1158 (11.36%)	113 (15.12%)	
High school graduate	120 (30.63%)	2440 (23.93%)	168 (22.49%)	
Some college	133 (33.90%)	3187 (31.25%)	226 (30.26%)	
College graduate or above	55 (13.98%)	2918 (28.61%)	193 (25.90%)	
Vigorous activity				
Yes	95 (24.24%)	2786 (27.32%)	228 (30.53%)	.2573
No	294 (75.24%)	7346 (72.03%)	514 (68.87%)	
Unable to do activity	2 (0.51%)	663 (0.65%)	45 (0.60%)	
Alcohol use				
Yes	290 (74.16%)	8733 (85.63%)	643 (86.14%)	<.001
No	101 (25.84%)	1465 (14.37%)	104 (13.86%)	
Smoking status				
Never	189 (48.38%)	4993 (48.96%)	355 (47.55%)	.0100
Former	83 (21.12%)	2763 (27.09%)	221 (29.60%)	
Current	119 (30.50%)	2443 (23.96%)	171 (22.85%)	
Hypertension history				
Yes	99 (25.24%)	2985 (29.27%)	268 (35.82%)	.0003
No	292 (74.66%)	7614 (70.73%)	479 (64.18%)	
Diabetes history				
Yes	30 (7.6%)	728 (7.14%)	74 (9.90%)	.0232
No	360 (92.03%)	9296 (91.16%)	659 (88.16%)	
Borderline	1 (0.37%)	174 (1.71%)	14 (1.94%)	

ABSI = A Body Shape Index, BMI = body mass index, PIR = poverty income ratio.

When comparing the characteristics of constipated and non-constipated participants, significant differences were observed. The proportion of female participants was significantly higher in the constipated group than in the non-constipated group (*P* < .05). Additionally, we found that a lower BMI was associated with a higher likelihood of constipation (*P* < .05).

### 4.2. The relationship between ABSI and constipation

The results of the multivariate logistic regression analysis examining the relationship between ABSI and constipation are shown in Table 2. In Model 1, a significant positive association was observed between ABSI and the incidence of constipation. However, this association was no longer significant after adjusting for gender, age, and race in Model 2. In Model 3, which adjusted for all covariates, a significant positive association between ABSI and constipation was reestablished (odds ratio [OR] = 265.1; 95% CI: 26.8–503.5; *P* = .029).

**Table 2:** Multiple logistic regression results: relationship between ABSI and bowel movement frequency categories.

	Model I OR (95% CI) *P* value	Model II OR (95% CI) *P* value	Model III OR (95% CI) *P* value
ABSI	568.6 (363.8, 773.5) <.0001	170.5 (−62.2, 403.1) .151	265.1 (26.8, 503.5) .029
Categories			
Constipation	−0.3 (−1.3, 0.6) .491	−0.7 (−1.7, 0.3) .161	−0.4 (−1.5, 0.6) .428
Normal bowel	3.1 (1.6, 4.5) <.001	1.2 (−0.4, 2.9) .138	1.8 (0.1, 3.5) .039
Diarrhea	8.0 (−1.7, 17.8) .105	9.6 (−1.7, 20.8) .095	6.3 (−5.4, 18.0) .290

Model I: no adjustments; Model II: adjusted for age, gender, and race; Model III: adjusted for age, gender, race, education, poverty-to-income ratio (PIR), height,alcohol use, smoking status, diabetes history, hypertension history, vigorous activity, protein intake, carbohydrate intake, dietary fiber intake, total fat intake, and moisture intake.

ABSI = A Body Shape Index, CI = confidence interval, OR = odds ratio.

We also performed additional regression analyses by categorizing participants into 3 groups based on bowel movement frequency. As shown in Table 2, the OR and its corresponding confidence interval (CI) revealed a significant positive correlation for the normal bowel group after adjusting for all covariates in Model 3. Specifically, the OR was 1.8 (95% CI: 0.1–3.5, *P* = .039), indicating a statistically significant difference between the groups. These results suggest a significant positive correlation between ABSI and bowel movement frequency within the range of 3 to 21 bowel movements per week.

### 4.3. Subgroup analysis

Comprehensive subgroup analyses were performed to evaluate the consistency of the association between ABSI and constipation across various demographic and clinical subgroups. These stratified analyses aimed to identify potential effect modifiers and provide a more detailed understanding of this relationship. As shown in Table 3, our results revealed a significant association between elevated ABSI and reduced bowel frequency, indicating that higher ABSI values were associated with an increased likelihood of constipation.

**Table 3:** Subgroup analysis of the association between ABSI and constipation.

Variable	OR (95% CI)	*P* value	*P* for interaction
Gender			
Male	692.3 (286.9, 1097.8)	.00	.02
Female	104.4 (−193.1, 402.0)	.49	
Race			
Mexican American	−13.2 (−980.1, 953.8)	.98	.94
Other Hispanic	84.7 (−1189.6, 1359.1)	.90	
Non-Hispanic White	234.1 (−44.9, 513.1)	.10	
Non-Hispanic Black	407.4 (−311.4, 1126.2)	.27	
Other Race	508.4 (−614.9, 1631.7)	.38	
Education			
<9th grade	−3.1 (−1150.0, 1143.8)	.99	.75
9–11th grade	8.2 (−676.0, 692.5)	.98	
High school graduate	330.0 (−154.1, 814.1)	.18	
Some college	144.2 (−292.7, 581.1)	.52	
College graduate or above	463.6 (22.9, 904.4)	.04	
Vigorous activity			
Yes	352.4 (−132.5, 837.3)	.15	.90
No	226.2 (−49.7, 502.1)	.10	
Unable to do activity	114.7 (−2734.8, 2964.2)	.94	
Alcohol use			
Yes	264.1 (7.4, 520.8)	.04	.97
No	252.6 (−280.3, 785.5)	.35	
Smoking status			
Never	250.3 (−88.6, 589.3)	.15	.78
Former	276.1 (−190.1, 742.2)	.25	
Current	457.3 (−32.3, 946.8)	.07	
Hypertension history			
Yes	535.2 (107.2, 963.3)	.01	.17
No	186.5 (−96.2, 469.2)	.20	
Diabetes history			
Yes	174.4 (−668.7, 1017.6)	.69	.73
No	304.2 (54.3, 554.1)	.02	
Borderline	−390.3 (−2202.3, 1421.6)	.67	

Analyses of subgroups that incorporate covariates adjusted for in Model 3.

95% CI = 95% confidence interval, OR = odds ratio.

Notably, within the male subgroup, ABSI showed a strong positive correlation with constipation (OR = 692.3; 95% CI: 286.9–1097.8; *P* = .0008). This robust association suggests that males with elevated ABSI may face significantly higher risks of developing constipation. These findings highlight the potential utility of ABSI as a predictive marker for bowel dysfunction, especially in specific demographic groups. Further research is needed to validate these findings across diverse populations and to explore the biological mechanisms that underlie this association.

### 4.4. Non-linear relationship between ABSI and constipation

**Figure 2.**
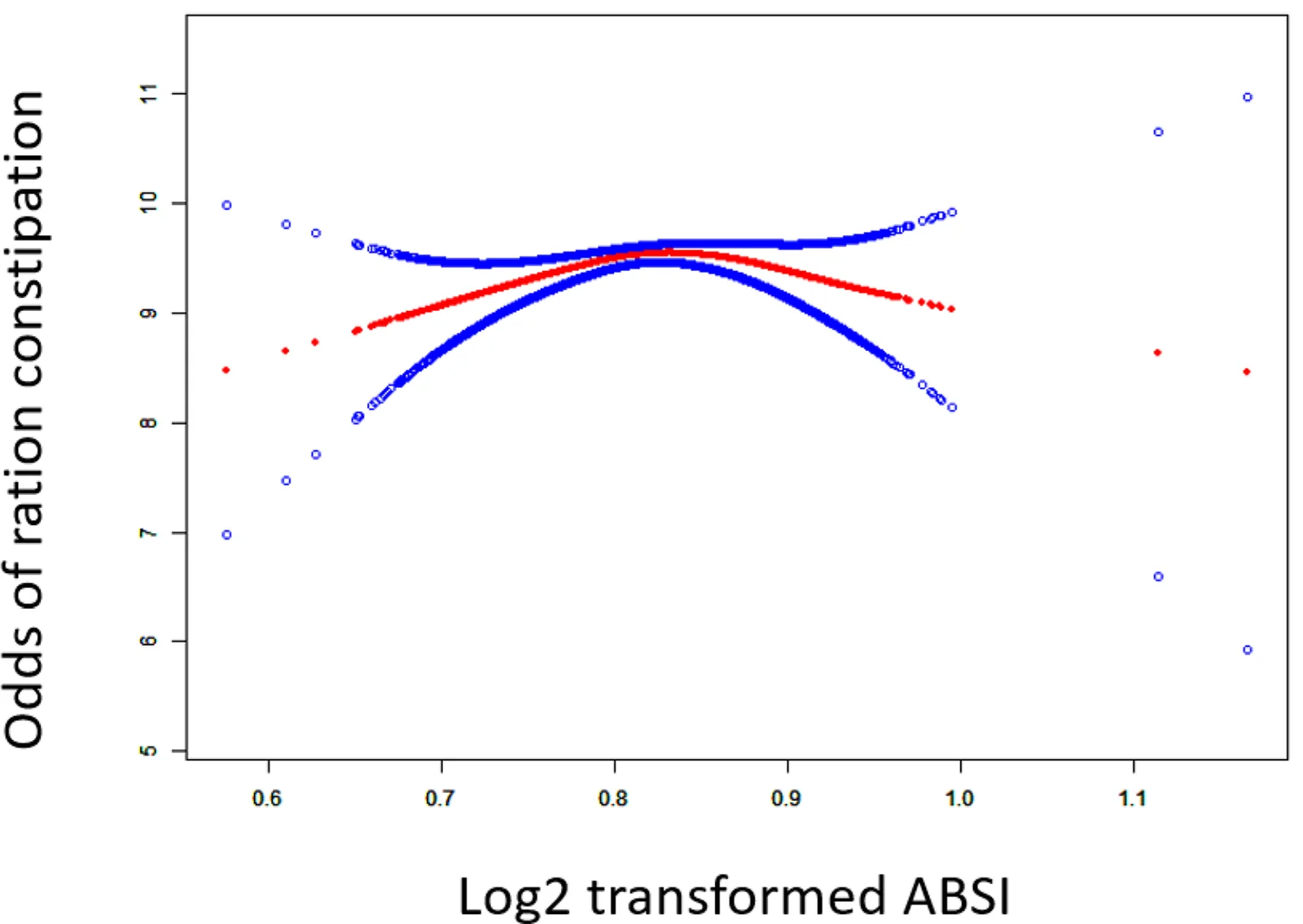
Association between ABSI and constipation. In the RCS model, age, gender, race, education, poverty-to-income ratio (PIR), height, alcohol use, smoking status, diabetes history, hypertension history, vigorous activity, protein intake, carbohydrate intake, dietary fiber intake, total fat intake, and moisture intake were adjusted. ABSI = A Body Shape Index, RCS = restricted cubic spline.

We further explored the relationship between ABSI and the risk of constipation, revealing a positive non-linear association before reaching an inflection point (ABSI*100 < 0.82) (Fig. 2).

Association between ABSI and constipation. In the RCS model, age, gender, race, education, PIR, height, alcohol use, smoking status, diabetes history,hypertension history, vigorous activity, protein intake, carbohydrate intake, dietary fiber intake, total fat intake, and moisture intake were adjusted.

## 5. Discussion

In this cross-sectional analysis of 11,336 participants, we identified a significant association between ABSI and constipation, with higher ABSI values linked to an increased risk of constipation, particularly in men, individuals with hypertension, and alcohol users. Our RCS analyses further revealed a significant non-linear relationship between ABSI and constipation. Specifically, when ABSI was below 0.0082, the incidence of constipation increased with rising ABSI levels. This association remained statistically significant even after adjusting for multiple confounders, suggesting that ABSI may serve as an important independent predictor of constipation.

Several potential mechanisms may explain the observed association between ABSI and constipation. From a physiological standpoint, central obesity, as indicated by elevated ABSI values, is associated with a chronic inflammatory state. This inflammation triggers the secretion of pro-inflammatory cytokines, such as interleukin-6, C-reactive protein, and tumor necrosis factor-α, which may disrupt the enteric nervous system and impair peristalsis, thereby contributing to constipation.^[14]^ Additionally, obese individuals often exhibit lower gut microbiota diversity,^[15]^ and studies have shown that obesity is associated with reduced levels of short-chain fatty acids (SCFAs), which are vital for gut health and overall metabolism. This altered gut environment can exacerbate constipation.

Moreover, central obesity may directly affect intestinal motility by increasing abdominal pressure. This mechanical pressure could lead to intestinal dysfunction, further worsening constipation.^[16]^ Obesity may also impact intestinal function through the neuroendocrine system, particularly the hypothalamic-pituitary-adrenal axis and the gut-brain axis, both of which are involved in regulating appetite, energy metabolism, and intestinal motility.^[14]^

In the subgroup analyses, the association between ABSI and constipation was more pronounced in men, individuals with higher education, alcohol drinkers, and those with a history of hypertension. The notably higher OR observed in the male subgroup can be attributed to several physiological and pathophysiological factors. Men typically accumulate more visceral fat compared to women, which is reflected in their higher ABSI values. This gender difference in fat distribution leads to various metabolic and inflammatory changes. Visceral fat is metabolically active and functions as an endocrine organ, secreting pro-inflammatory cytokines.^[17]^ These cytokines induce a chronic low-grade inflammatory state that not only promotes fat deposition but also disrupts normal gastrointestinal function, creating a self-perpetuating cycle of inflammation and metabolic dysregulation.

Furthermore, the inflammatory environment associated with visceral fat accumulation affects the gut microbiota, altering its composition and function. Dysbiosis impacts the production of metabolites, such as SCFAs, which are essential for regulating intestinal cell signaling and gastrointestinal motility. A reduction in SCFA production or alterations in signaling pathways can impair peristalsis, thus increasing the risk of constipation.^[18]^ Studies have also shown that women generally have higher gut microbiota diversity compared to men, with a greater enrichment of metabolically protective organisms, such as ‘Akkermansia muciniphila’and “Alistipes shahii,” which support normal metabolism and intestinal health.^[19]^

Collectively, these interrelated physiological mechanisms highlight how gender differences in visceral fat accumulation, along with the associated inflammatory and metabolic effects, amplify the relationship between ABSI and constipation.

The significant association between ABSI and constipation in hypertensive patients may be influenced by comorbidities, particularly the effects of hypertension-related medications (e.g., calcium channel blockers). Studies have shown that high blood pressure typically leads to sympathetic hyperactivity, which may reduce colonic motility and contribute to constipation. Furthermore, some antihypertensive medications, such as calcium channel blockers and diuretics, have been shown to exacerbate constipation.^[20,21]^

Additionally, alcohol consumption may intensify the effects of ABSI on constipation by altering gut flora and exacerbating the inflammatory response, further disrupting gastrointestinal function.

ABSI, as a simple and easily measurable indicator, can be used to identify individuals at high risk of constipation, particularly among men and those with a history of hypertension. However, large-scale studies examining the relationship between ABSI and constipation risk are still lacking. Our study also has some limitations. Firstly, as a cross-sectional study, it cannot establish a causal relationship between ABSI and constipation. Secondly, constipation was defined solely based on bowel movement frequency, which may not fully capture other characteristics of constipation, such as stool traits, potentially introducing bias. Additionally, the narrow range of ABSI values may limit the interpretation of its effects.

Future research should include longitudinal studies to clarify the causal relationship between ABSI and constipation. Further exploration of the modifying effects of gender, alcohol consumption, and hypertension on the ABSI-constipation association is also warranted. Moreover, investigating the correlation between ABSI and other constipation indicators, such as stool consistency and gut transit time, could provide deeper insights into this relationship.

## 6. Conclusion

In conclusion, this large-scale study provides novel insights into the relationship between ABSI and constipation. The findings suggest that ABSI may serve as a valuable predictor of constipation, particularly in specific demographic groups, such as males and individuals with hypertension. Furthermore, intervention studies focusing on body shape and related factors, including dietary modifications, physical activity, and weight management programs, are crucial to assess their effectiveness in reducing the risk of constipation. These interventions should address the multifaceted nature of the relationship between ABSI and constipation, considering both individual and population-level health outcomes. By doing so, more personalized and effective strategies can be developed to prevent and manage constipation, ultimately improving the quality of life for individuals affected by this condition.

## Author contributions

**Conceptualization:** Canjie Wei.

**Data curation:** Canjie Wei, Hongling Huang.

**Formal analysis:** Yangtao Chen.

**Funding acquisition:** Jing Wang.

**Investigation:** Yameng Xie, Xun Wang.

**Methodology:** Yangtao Chen, Zhaochu Wang, Xun Wang.

**Resources:** Hongling Huang.

**Software:** Yangtao Chen, Zhaochu Wang, Yameng Xie.

**Supervision:** Zhaochu Wang, Yameng Xie, Xun Wang, Hongling Huang.

**Validation:** Zhaochu Wang.

**Writing – original draft:** Canjie Wei.

**Writing – review & editing:** Jing Wang.
